# Accurate Prediction of Total PlGF (Placental Growth Factor) From Free PlGF and sFlt-1 (Soluble Fms-Like Tyrosine Kinase-1): Evidence for Markedly Elevated PlGF Levels in Women With Acute Fatty Liver of Pregnancy

**DOI:** 10.1161/HYPERTENSIONAHA.121.17258

**Published:** 2021-06-28

**Authors:** Rugina I. Neuman, Langeza Saleh, Koen Verdonk, Anton H. van den Meiracker, Henk Russcher, Herold J. Metselaar, Willy Visser, A.H. Jan Danser

**Affiliations:** 1 Division of Pharmacology and Vascular Medicine, Department of Internal Medicine (R.I.N., L.S., K.V., A.H.v.d.M., W.V., A.H.J.D.), Erasmus MC University Hospital, Rotterdam, the Netherlands.; 2 Department of Gynecology and Obstetrics (R.I.N., W.V.), Erasmus MC University Hospital, Rotterdam, the Netherlands.; 3 Department of Clinical Chemistry (H.R.), Erasmus MC University Hospital, Rotterdam, the Netherlands.; 4 Department of Gastroenterology & Hepatology (H.J.M.), Erasmus MC University Hospital, Rotterdam, the Netherlands.

**Keywords:** biomarkers, fatty liver, HELLP syndrome, placental growth factor, pregnancy

## Abstract

Acute fatty liver of pregnancy (AFLP) is characterized by elevated circulating sFlt-1 (soluble Fms-like tyrosine kinase-1), although the free circulating levels of its ligand, PlGF (placental growth factor) are not decreased. Here, we hypothesized that women with AFLP exhibit elevated PlGF production in comparison to women with preeclampsia or hemolysis elevated liver enzymes and low platelet count syndrome. Making use of the well-known mathematical formulas describing drug-receptor interactions, we established that serum total PlGF could be accurately predicted from sFlt-1 and free PlGF levels (n=42; mean calculated K_D_ of 50 pmol/L), yielding similar values as the previously published method of thermal dissociation of the sFlt-1-PlGF complexes (*r*=0.94, *P*<0.0001). We found that median levels of free PlGF were significantly lower in women with preeclampsia (n=13; 117pg/mL) or hemolysis elevated liver enzymes and low platelet count syndrome (n=12; 59 pg/mL) compared with women without preeclampsia (n=11; 349pg/mL, *P*<0.0001). In contrast, median total PlGF did not differ between women with no preeclampsia, preeclampsia, and hemolysis elevated liver enzymes and low platelet count syndrome (354 versus 435 versus 344pg/mL), whereas it was markedly elevated in AFLP compared with all groups (2054 pg/mL, *P*<0.0001). Furthermore, in AFLP, both sFlt-1 and total PlGF declined rapidly postdelivery, with significantly higher predelivery total PlGF (n=12; median, 2054 pg/mL) than postpartum levels (n=14; median, 163pg/mL, *P*<0.0001), suggesting that in AFLP, PlGF is largely placenta-derived. Collectively, our findings indicate that like sFlt-1, PlGF production is significantly upregulated in AFLP, mainly originating from the placenta. Importantly, total PlGF can now be easily calculated from already available free PlGF and sFlt-1 levels, allowing subsequent evaluation of other groups in whom PlGF is altered.

Acute fatty liver of pregnancy (AFLP) is a severe liver disorder unique to pregnancy, typically occurring after 30 weeks’ gestation.^1^ Although uncommon, with an estimated global incidence of 1 in 7000 to 15 000 pregnancies, AFLP is a life-threatening disease for both mother and child.^1^ The exact pathogenesis of AFLP remains unclear, but it is generally believed that mitochondrial oxidation defects of the fetus or placenta lead to a buildup of free fatty acids in the maternal blood and hepatocytes, subsequently causing the detrimental manifestations of the disorder.^1^ The most prominent characteristic of AFLP is the presence of hepatic dysfunction, reflected by complications such as hypoglycemia, coagulopathy, and renal failure.^1,2^ Despite these distinctive features, differentiating AFLP from other liver diseases of pregnancy, particularly the hemolysis elevated liver enzymes and low platelet count (HELLP) syndrome, remains a challenge in clinical practice.^2,3^ An important factor underlying this difficulty might be that up to 20% of women with AFLP are also diagnosed with preeclampsia, a condition characterized by hypertension in the second half of pregnancy along with proteinuria or signs of maternal organ damage, including HELLP syndrome.^1,4,5^ Whether preeclampsia and AFLP are merely associated with one another or belong to a spectrum of the same disorder, as some have suggested,^6,7^ is uncertain.

During preeclampsia, poor placentation triggers the excessive release of the sVEGFR (soluble vascular endothelial growth factor receptor; also known as sFlt-1 [soluble Fms-like tyrosine kinase-1]), which binds its free circulating ligands VEGF and PlGF (placental growth factor).^8,9^ The ensuing angiogenic imbalance is thought to contribute significantly to the clinical manifestations of this disorder.^10^ Interestingly, our group has recently discovered that women with AFLP also display increased levels of sFlt-1 in their maternal circulation.^11^ Yet, despite the markedly elevated sFlt-1 levels, women with AFLP exhibited higher free levels of PlGF, in contrast to what is observed in HELLP syndrome.^11^

Based on our previous observations, we hypothesized that women with AFLP display elevated PlGF production in comparison to preeclampsia/HELLP syndrome. Recently, Lecarpentier et al^12^ established a novel method to measure total PlGF in maternal blood because commercial immunoassays only detect the unbound (free) form of PlGF.^12^ Hence, in the present study, we intended to (1) validate this method in a small population of women with a low and high sFlt-1/free PlGF ratio; (2) determine whether a more simple approach, by calculating total PlGF mathematically from sFlt-1 and free PlGF, would prove equally reliable as thermal dissociation; and (3) compare serum total PlGF levels in women with AFLP to women with no preeclampsia, preeclampsia or HELLP syndrome. In addition, we explored the origin of the angiogenic markers in AFLP and whether they could derive from the placenta.

## Methods

All data and supporting materials have been provided within the published article.

### Human Participants

#### Participants With AFLP

Human serum samples from a database of women with singleton pregnancies who had a clinical diagnosis of AFLP at the Erasmus Medical Center, Rotterdam, the Netherlands between 2005 and 2020 were utilized. Serum samples were collected at the time of AFLP diagnosis (both during and after pregnancy) and later archived at −80 °C as part of routine care. Residual material with enough volume for the analysis of sFlt-1, free and total PlGF was collected for this study if the patients did not object against use of this material. The use of these samples for the purposes of this study was exempted from approval by the local institutional Medical Ethics Committee according to the Dutch Medical Research with Human Participants Law (MEC-2020-0668). All laboratory assays were undertaken masked to the clinical diagnosis. Clinicians had no knowledge of the angiogenic measurements at time of AFLP diagnosis. The diagnosis of AFLP was suspected when a pregnant woman had symptoms of nausea, vomiting, fatigue, and anorexia at the end of the second or third trimester in combination with jaundice and elevated liver enzymes. The diagnosis of AFLP was confirmed when a woman fulfilled ≥6 out of 15 Swansea criteria,^13^ and the treating physician found the clinical diagnosis of AFLP as most likely in comparison to other disorders, such as HELLP syndrome. Pregnancy characteristics and outcome were obtained from the digital medical files.

#### Participants With No Preeclampsia, Preeclampsia, or HELLP Syndrome

We aimed to compare all women with AFLP to 12 gestational age (GA) matched women with either no preeclampsia, confirmed preeclampsia, or HELLP syndrome, given that the values of sFlt-1, free, and total PlGF alter with advancing gestation.^14^ Available residual serum samples from these three groups were collected from a previously conducted prospective cohort study in which the sFlt-1/free PlGF ratio was measured in singleton pregnancies with suspected or confirmed preeclampsia, between 2013 and 2016 at 3 hospitals in the Netherlands. This study was approved by the research ethics committee (MEC-2013-202), and written informed consent was obtained from all participants. Venous blood was taken at study entry only and was stored at −80 °C until analysis, which was conducted at the end of the study, to avoid influence on decision making of the obstetricians.^9^ Preeclampsia was defined as the presence of new-onset hypertension (systolic blood pressure of ≥140 mm Hg or diastolic blood pressure of ≥90 mm Hg) and proteinuria (protein-to-creatinine ratio ≥30 mg/mmol or ≥300 mg/24 hours or 2+ dipstick) at or after 20 weeks’ gestation, according to the 2001 International Society for the Study of Hypertension in Pregnancy definition, which was in effect at the time of study initiation.^15^ HELLP syndrome was defined as a reduction of platelet count <100×10^9^/L, an elevation of ALT (alanine aminotransferase) or AST (aspartate aminotransferase) 2-fold the upper limit of normal, and an elevated LDH (lactate dehydrogenase; 2-fold the upper reference limit or >650 IU/L) according to the International Society for the Study of Hypertension in Pregnancy 2013 definition.^16^ Women who had a partial HELLP syndrome (≥2 of the HELLP criteria) at time of blood sampling but later developed HELLP syndrome were also considered as HELLP syndrome. Women who were initially suspected of preeclampsia but did not fulfill the diagnosis of gestational hypertension, preeclampsia, or HELLP syndrome throughout their pregnancy were defined as no preeclampsia.

For the validation studies, we selected serum samples from patients with a low angiogenic imbalance (sFlt-1/free PlGF ratio ≤38) versus a high angiogenic imbalance (sFlt-1/free PlGF ratio ≥85) from the abovementioned cohort study. These cutoff values were previously reported to predict the short-term absence of preeclampsia (sFlt-1/free PlGF ratio ≤38)^17^ or a high risk of preeclampsia-related adverse outcomes (sFlt-1/free PlGF ratio ≥85).^18^ All pregnancy characteristics and outcome were obtained from digital medical files.

#### Participants With Acute Liver Failure

Available serum samples from nonpregnant women with acute liver failure requiring immediate liver transplantation at the Erasmus Medical Center, Rotterdam, the Netherlands, were selected at random, for the measurement of sFlt-1 and total PlGF. The use of these samples for this study was exempted from approval by the local institutional Medical Ethics Committee according to the Dutch Medical Research with Human Participants Law (MEC-2014-060).

### Measurement of sFlt-1, Free PlGF, and Total PlGF

#### Validation Studies

rhPlGF (human recombinant PlGF;

264-PGB-010/CF, R&D Systems, Minneapolis, MN) was dissolved in PBS containing 0.1% BSA (PBS). This solution was mixed 1:3 with either PBS or serum to reach a final rhPlGF concentration of 1693 pg/mL. For comparison, serum samples were also mixed 1:3 with PBS not containing rhPlGF. Serum was obtained from pregnant patients with either a low (≤38) or a high (≥85) sFlt-1/free PlGF ratio. All samples (rhPlGF-containing PBS, serum with PBS, and serum with rhPlGF-containing PBS) were incubated for 30 minutes at room temperature to allow rhPlGF to bind to sFlt-1. Next, sFlt-1 and PlGF were measured in all samples before and after heating.

#### Heating Procedure and Biochemical Measurements

For the thermal dissociation of all sFlt-1-PlGF complexes, serum samples were placed in a heating block at 70 °C for 10 minutes, as described by Lecarpentier et al.^12^ Measurements of sFlt-1 and PlGF before and after heating were performed using the automated Elecsys immunoassay from Roche Diagnostics (Cobas 6000, e-module; Rotterdam, the Netherlands).

### Calculation of Total PlGF from Free PlGF and sFlt-1

In blood plasma, an equilibrium exists between free sFlt-1, PlGF, and their complex, described by the following equation: [sFlt-1]+[PlGF]⇌[sFlt-1−PlGF]. This equals classic drug-receptor interaction, with [sFlt-1] resembling the total number of receptors [R]_total_, free PlGF ([PlGF]_free_, ie, the PlGF level measured without heating) resembling the amount of nonreceptor-bound drug [D], and [sFlt-1-PlGF] resembling the number of drug-occupied receptors [DR]. The dissociation constant K_D_=[D]×[R]/[DR]. Given that [R]_total_=[R]+[DR], this formula can be rewritten as K_D_=−[D]+[D]×[R]_total_/[DR]. Since [DR] can be calculated by subtracting [PlGF]_total_ (ie, the PlGF level obtained after heating) from [PlGF]_free_ and considering that the molecular weights of PlGF and sFlt-1 are 34 and 100 kD, respectively, it is now possible to calculate K_D_. This approach was followed in all samples where free and total PlGF levels were available, with total PlGF being higher than free PlGF, allowing us to calculate a mean K_D_ on the basis of 42 samples. With this K_D_ we were able to predict [PlGF]_total_ by first calculating [DR], that is, the amount of PlGF bound to sFlt-1, as follows:


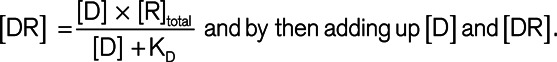


Since [D]=[PlGF]_free_, and [R]_total_=[sFlt-1], this translates to


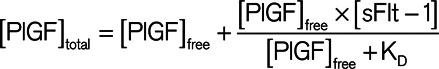


### Statistical Analysis

Data are presented as median (interquartile range) or number (percentage). To evaluate whether continuous variables had a normal distribution, the Shapiro-Wilk normality test was used. To compare groups, the Student *t* test or Mann-Whitney *U* test in case of non-normally distributed data were applied. For the comparison of continuous variables between >2 groups, 1-way ANOVA, or Kruskal-Wallis test, in the case of nonparametric distributions was applied, with a Dunnet or Bonferroni correction for multiple testing. Spearman Rho was applied to calculate correlation coefficients. A *P* value of <0.05 was considered to be statistically significant. Statistical analysis was performed with GraphPad Prism (version 8.0, La Jolla, CA) and SPSS (version 25.0, SPSS Chicago, IL) on Windows.

## Results

### Thermal Dissociation of sFlt-1-PlGF Complex Using Recombinant Human PlGF

**Figure 1.**
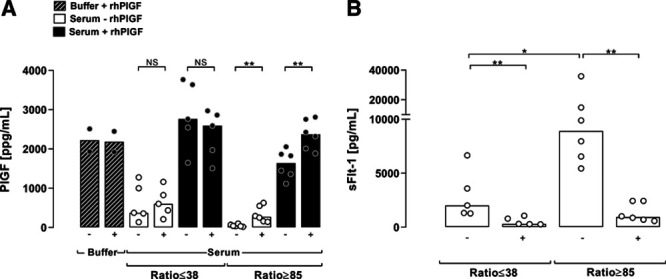
**Protein level measurements performed before and after heating.** PlGF (placental growth factor; **A**) and sFlt-1 (soluble Fms-like tyrosine kinase-1; **B**) levels in buffer (n=2) or serum measured before [−] and after [+] heating at 70 oC for 10 min, with or without the addition of rhPlGF (human recombinant PlGF; mixed 1:3 in either PBS or serum to reach a final rhPlGF concentration of 1693 pg/mL). Serum was obtained from pregnant women with an sFlt-1/free PlGF ratio ≤38 (n=5) or ≥85 (n=6). Data are presented as individual values and median (bar). *P<0.05; **P<0.01.

To confirm the method of thermal dissociation, we added rhPlGF (recombinant human PlGF) to serum samples from pregnancies with a low (≤38) or high sFlt-1/free PlGF ratio (≥85), and measured sFlt-1 and PlGF before and after heating, with and without rhPlGF. Five participants with a ratio ≤38 and 6 participants with a ratio ≥85 were evaluated, whose clinical characteristics and pregnancy outcomes are shown in Table 1. In participants with a ratio ≤38, serum PlGF levels marginally (*P*=nonsignificant) increased after thermal dissociation. Heating did not affect the detection of rhPlGF, and also after adding rhPlGF to serum we did not detect higher levels after heating than before (Figure 1A).

**Table 1.**
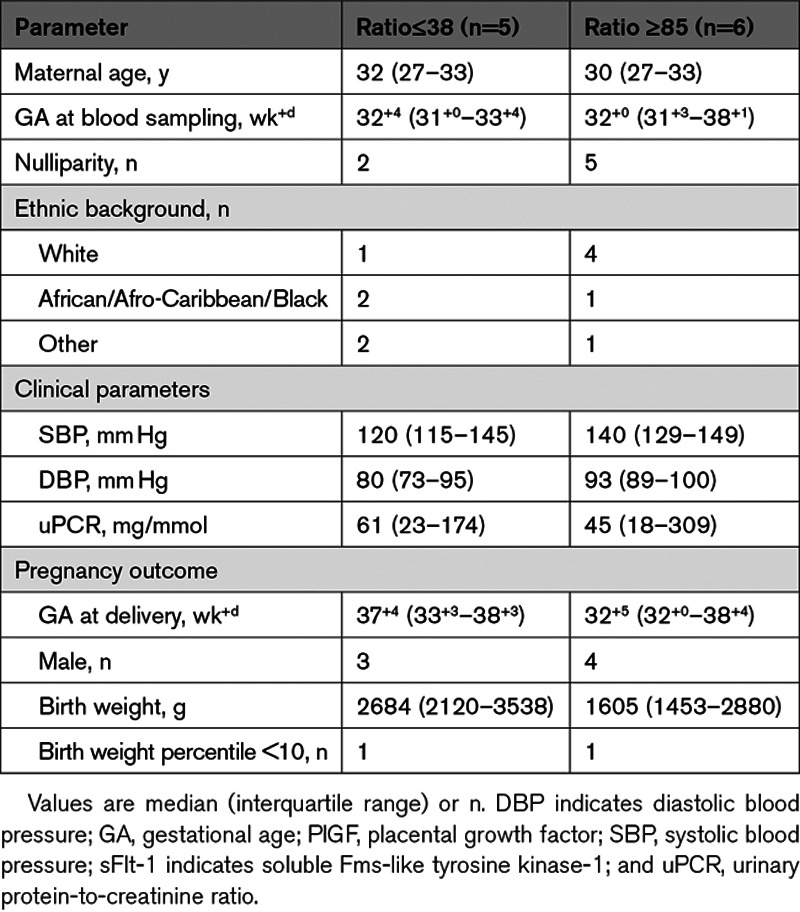
Participant Characteristics at Time of Blood Sampling of the Validation Cohort Based on sFlt-1/Free PlGF Ratio

In contrast, in participants with a ratio ≥85, serum PlGF levels were significantly higher after thermal dissociation than before (270 versus 36 pg/mL). Furthermore, when adding a fixed amount of rhPlGF to serum in this group (ratio ≥85), the levels detected in the absence of heating were significantly lower than the amount that was added (1634 versus 2221 pg/mL). This confirms binding by sFlt-1. Subsequent heating allowed the detection of a PlGF amount that equaled the sum of the added level and the endogenous level (2372 pg/mL; Figure 1A). Finally, as expected, the sFlt-1 levels were much higher in the ratio ≥85 group, whereas heating greatly diminished the amount of sFlt-1 that could be detected. These data confirm that heating selectively destroys sFlt-1, without affecting PlGF (Figure 1B), thus allowing the quantification of total PlGF.

### sFlt-1, Free, and Total PlGF in Women With AFLP

**Figure 2.**
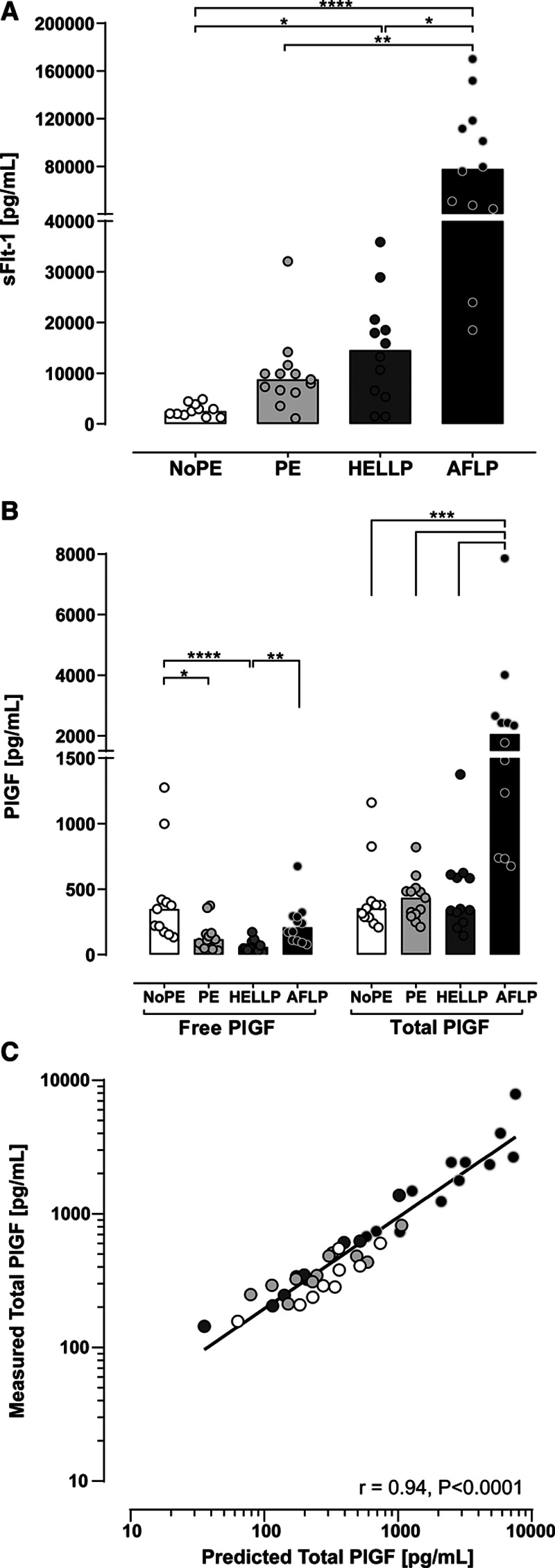
**Serum protein levels according to clinical diagnosis.** Antepartum serum sFlt-1 (soluble Fms-like tyrosine kinase-1; **A**) and PlGF (placental growth factor; free and total; **B**) levels in women with no preeclampsia (no PE; n=11), preeclampsia (PE; n=13), hemolysis (elevated liver enzymes and low platelet count (HELLP) syndrome (n=12) and acute fatty liver of pregnancy (AFLP; n=12). Data are presented as individual vales and median (bar). *P<0.05; **P<0.01, ***P<0.001 and ****P<0.0001. **C**, Correlations between measured and predicted total PlGF according to no PE (white circles), PE (light gray circles), HELLP (dark gray circles) and AFLP (black circles; n=42, *r*=0.94, *P*<0.0001).

Twelve women with AFLP were compared with women with no preeclampsia (n=11), confirmed preeclampsia (n=13), or HELLP syndrome (n=12). Because most of the women with HELLP syndrome from the previously conducted cohort^9^ had a lower GA at blood sampling, we were unable to adequately match the AFLP group to women with HELLP syndrome at a similar GA. All participant characteristics and pregnancy outcomes according to clinical diagnosis are shown in Table 2. Women with AFLP displayed higher median sFlt-1 levels (77 762 [45 044–116 657] pg/mL) compared with women with no preeclampsia, preeclampsia, and HELLP syndrome (2518 [1744–3903], 8772 [6410–10 736] and 14 572 [5641–20 056] pg/mL, respectively; Figure 2A). When comparing free PlGF levels, women diagnosed with preeclampsia and HELLP syndrome displayed lower PlGF values than women with no preeclampsia, whereas free PlGF levels in women with HELLP syndrome were also significantly decreased in comparison to women with AFLP (59 [39–97] pg/mL versus 208 [106–293] pg/mL; Figure 2B). In contrast, total PlGF levels (measured after thermal dissociation) did not differ between women with no preeclampsia, preeclampsia, and HELLP syndrome, whereas they were significantly increased in AFLP in comparison to all groups (Figure 2B). None of the women were on heparin treatment at time of blood sampling.

**Table 2.**
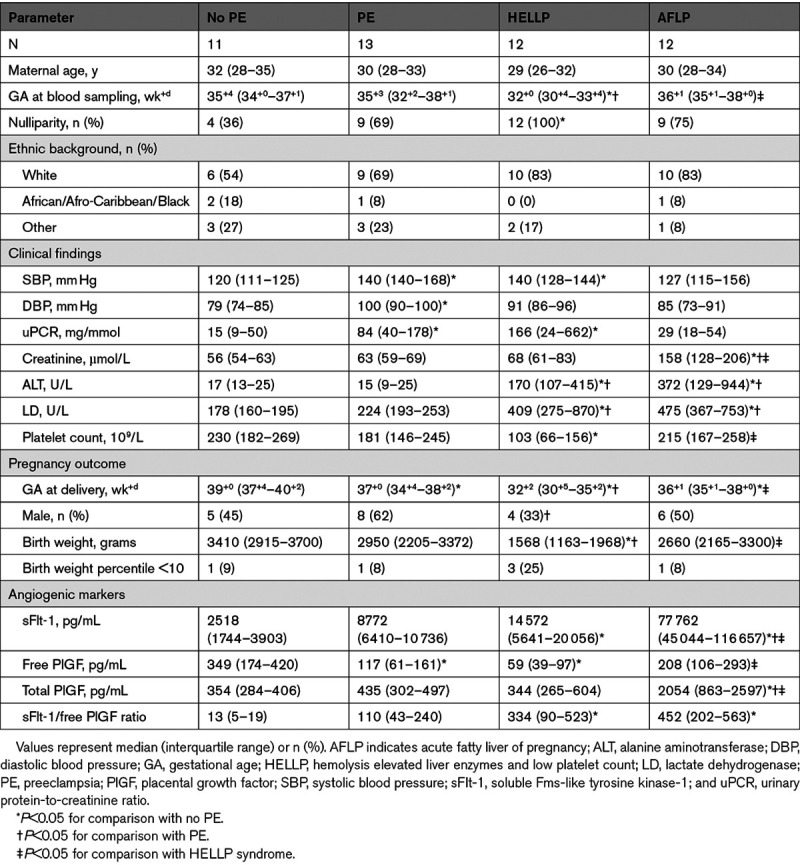
Pregnancy Characteristics of All 48 Participants According to Clinical Diagnosis

### Prediction of Total PlGF and Comparison With Its Measured Value

K_D_ was calculated in all samples in which we determined both free and total PlGF. This resulted in an average K_D_ (±SEM) value of 50±6.4 pmol/L. Next, we predicted total PlGF in each sample making use of this K_D_ value, and compared it with the actually measured total PlGF value. As can be seen in Figure 2C, this yielded a relationship that was not different from the line of identity (*r*=0.94, *P*<0.0001; Figure 2C).

### Antepartum Versus Postpartum Levels of sFlt-1, Total PlGF, and ALT

**Figure 3.**
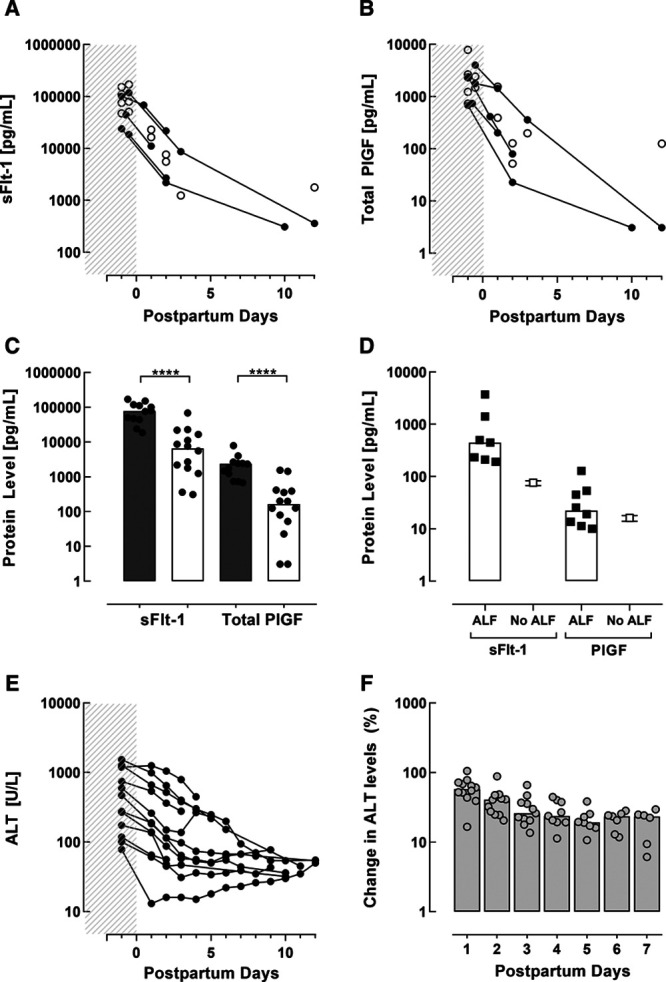
**Protein levels pre- and postpartum, and in patients with acute liver failure.** **A** and **B**, sFlt-1 (soluble Fms-like tyrosine kinase-1) and total PlGF (placental growth factor) values in women with acute fatty liver of pregnancy (AFLP) determined before (shaded area; n=12) or after delivery (n=11, 14 measurements total), according to the number of postpartum days. Values are depicted as single (○) or repeated measurements (•). **C**, Median and individual levels of sFlt-1 or total PlGF in all women with AFLP before delivery (gray bars; n=12) and after delivery (white bars; n=14). Antepartum blood was taken ≤2 d before delivery, whereas postpartum blood was drawn at 0–12 d postpartum. ***P<0.001. **D**, Median and individual levels of sFlt-1 (n=7) and total PlGF (n=8) in nonpregnant women with acute liver failure () in comparison to reference values (median and interquartile range) in healthy nonpregnant women for sFlt-1 and free PlGF (□). **E**, Alanine aminotransferase (ALT) values of all 12 women with AFLP determined before (shaded area) and after delivery, according to the number of postpartum days. **F**, Postpartum ALT values of all 12 women with AFLP depicted as percentages of antepartum values in the first week after delivery.

In 12 women with AFLP, measurements of sFlt-1 and total PlGF were performed antepartum. In 5 of these 12 women, postpartum levels were additionally measured, and in 3 of these 5, postpartum measurements were performed at 2 separate time-points. Additionally, there were 66 women with AFLP in which the values of sFlt-1 and total PlGF were determined postpartum only. Figure 3A and 3B show that the levels of sFlt-1 decreased by >80% within 2 days after delivery, whereas for total PlGF, the drop was even larger, with <10% remaining after 2 days. This pattern was fully confirmed when simply comparing the median levels before delivery with those after, irrespective of the sampling moment (Figure 3C). The postpartum course of ALT values in AFLP patients was much more gradual (Figures 3E and 3F).

### sFlt-1 and Total PlGF Levels in Participants With Acute Liver Failure in Need of Liver Transplantation

Median levels for sFlt-1 were available in 7 patients, whereas total PlGF was measured in 8 patients with liver failure in need of liver transplantation. The clinical characteristics of these patients are shown in Table 3. All patients were female with a median (interquartile range) of 28 (24–31) years. The most common reason for liver transplantation was toxic or drug-induced liver failure (n=4). Median total PlGF levels of these patients were 22 (12–51) pg/mL, which is comparable to the reference values for free PlGF in healthy nonpregnant women (16 [14–18] pg/mL; Figure 3D).^19^ In contrast, median sFlt-1 levels (446 [211–1414] pg/mL) were ≈6-fold above the normal range in healthy nonpregnant women (76 [67–84] pg/mL).

**Table 3.**
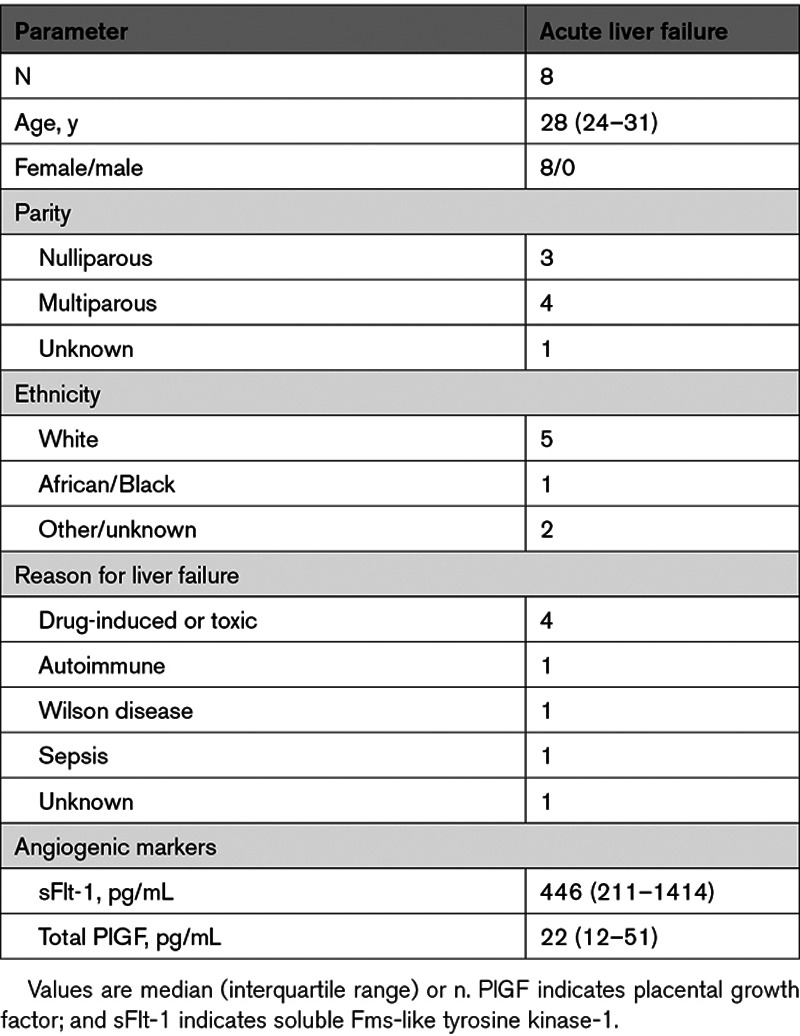
Characteristics of Nonpregnant Women With Acute Liver Failure

## Discussion

In the present study, we established that total PlGF levels in serum can be accurately calculated from the sFlt-1 and free PlGF levels, making use of the well-known mathematical formulas describing drug-receptor interaction. This approach yielded the same levels as the previously published method of thermal dissociation. We confirmed that total PlGF in preeclampsia is similar to that in uneventful pregnancy, although this is also true for HELLP. Yet, in AFLP, maternal total PlGF levels were greatly elevated, both versus women with preeclampsia and HELLP syndrome. Furthermore, in AFLP, total PlGF and sFlt-1 declined rapidly after delivery, suggesting the placenta as their most likely source.

A potential role for sFlt-1 and PlGF in the pathophysiology of AFLP had not been suggested until recently, when our group and others^11,20^ noticed abundantly high circulating sFlt-1 levels in women with this disorder. sFlt-1 is a well-recognized feature of preeclampsia, in which its dramatic rise causes a significant reduction of free circulating PlGF levels.^10^ Although a decrease in placental PlGF production has also been suggested to contribute to the low PlGF levels in preeclamptic disease,^21,22^ we observed no differences in total PlGF when comparing women without preeclampsia, preeclampsia, or HELLP syndrome in the current study. This agrees with the view of Lecarpentier et al^12^ that decreased free PlGF levels in preeclampsia / HELLP are a consequence of higher sFlt-1 rather than decreased PlGF production of the placenta.^12^ With this perspective in mind, our previous observation that despite greater sFlt-1 elevation, free PlGF levels were not reduced in women with AFLP in comparison to women with preeclampsia, was quite surprising.^11^ Our present finding that total PlGF levels are significantly raised in women with AFLP now explains this observation.

Obviously, a key question is whether an elevated production or impaired metabolism accounts for the dramatic increases of PlGF in AFLP. Because the relatively small PlGF (MW 34 kD) can readily cross the glomerular filtration barrier, one could hypothesize that its elimination is compromised as renal function declines in AFLP. The observation that total PlGF in women with this disorder decreased rapidly after delivery, despite the persistence of elevated serum creatinine (data not shown), does not support this argument. Another possibility is that the ensuing liver injury and inflammation in AFLP drive increased PlGF production either in the liver or elsewhere. However, our observation that total PlGF is diminished by >90% within 2 days postpartum, whereas the liver recovers more gradually after delivery, indicates that a placental origin or placenta-stimulated PlGF synthesis somewhere else is more probable. Consistent with this hypothesis, serum total PlGF levels in nonpregnant patients with acute liver failure were roughly comparable to the free PlGF levels observed in healthy controls (median [interquartile range], 22 [12–51] versus 16 [14–18] pg/mL). Conversely, their sFlt-1 values were 6-fold higher in relation to the reference values of healthy nonpregnant women (76 [67–84] pg/mL). This suggests that liver failure might contribute, at least in part, to the higher sFlt-1 values observed in AFLP, but not to the higher PlGF levels. A partial nonplacental origin of sFlt-1 might also explain why in AFLP women the drop in sFlt-1 levels after delivery was more modest in comparison to that of total PlGF. In both preeclampsia and HELLP, as we have demonstrated before, the opposite is true: sFlt-1 falls by >90% within 2 days, while free PlGF levels off at around 30% to 40% of its predelivery concentrations. Yet, postpartum, the free PlGF levels reach the same nadir in all groups and resemble the total PlGF levels. The simplest explanation of our findings is therefore that the stronger total PlGF drop in AFLP (exceeding that of sFlt-1) is due to its very high predelivery levels.

The exact contribution of the different PlGF isoforms (1–4) to the levels of total PlGF is unknown. Previous studies have indicated that PlGF-1 and PlGF-2 are the main isoforms found in maternal blood,^23,24^ whereas commercial assays mainly measure free PlGF-1. Although differences between assay results may in part be due to different degrees of cross-reactivity, the serum levels of PlGF-1 and PlGF-2 are highly correlated in both normal and pathological pregnancies in all 3 trimesters.^25^ This supports their common origin and control mechanisms. It has therefore been suggested that knowledge on the precise contribution of these isoforms may not be of added clinical significance.^25^

Accounting for other sFlt-1 binding ligands, such as VEGF, might further improve the accuracy of the predicted total PlGF levels. Ideally, a competitive binding model is set up that takes into account the interaction of all potential binding partners (in particular the various isoforms of both VEGF and PlGF) with sFlt-1. This would require a wide range of assays to measure all these binding partners, as well as knowledge on their affinities for sFlt-1. In reality, we were able to accurately predict total PlGF from sFlt-1 and free PlGF in pregnant women with a considerable variation in clinical background. This would argue against huge variation in VEGF (and thus its capacity to occupy sFlt-1-binding sites) between these conditions. Moreover, current assays provide the levels of sFlt-1 and PlGF only, and thus a simple model with these 2 readily available parameters remains preferred.

The limitations of our study must be addressed. Although the number of women evaluated with AFLP remains limited, this reflects the rarity of this disorder. In addition, we were unable to find women with HELLP syndrome with a similar GA as the women with AFLP. However, if anything, one would expect free PlGF levels to be further decreased in women with HELLP syndrome at a later GA because free PlGF levels start declining from 29 to 32 weeks’ gestation until the end of pregnancy.^14^ Lastly, it should be taken into account that the pregnancies of women defined as no preeclampsia were not entirely healthy, which might have influenced the interpretation of total PlGF in this population.

## Perspectives

At present, it is not known why PlGF production would be so much increased in AFLP. Oxidative stress in placental mitochondria has already been observed in AFLP,^26^ and this is widely accepted to upregulate sFlt-1.^10,27^ Moreover, the poor uteroplacental perfusion as a consequence of liver failure and the ensuing hypovolemia in AFLP, is likely to have the same consequence.^2^ Yet, in AFLP, unlike preeclampsia,^10^ there is no evidence for abnormal placental development. Thus, one plausible theory might be that in AFLP the normal placenta is still able to counteract the increases of sFlt-1 by massively upregulating PlGF. As a consequence, the free PlGF levels in this disorder are in the normal pregnancy range. An alternative scenario could be that the inflammatory response and endothelial disruption following liver injury trigger the release of sFlt-1 and PlGF. Clearly, potential stimuli of sFlt-1 or PlGF, like proinflammatory cytokines and free fatty acids, are worth exploring in AFLP, if possible in liver and placental tissue.

Our study is the first to present a simple mathematical approach to obtain the total PlGF levels. Not surprisingly, our observed K_D_ falls within the binding affinity reported for both PlGF and VEGF in relationship to the Flt-1 receptor (≈20–200 pmol/L).^28,29^ Consequently, total PlGF can now be easily calculated from already available free PlGF and sFlt-1 levels, allowing the subsequent evaluation of other groups in whom PlGF might be upregulated or downregulated, for instance in preeclamptic women with intrauterine growth restriction. In addition, the increased total PlGF concentrations might aid in distinguishing AFLP from other liver disorders of pregnancy, particularly HELLP syndrome. To evaluate this, future studies should firstly validate our findings in a separate cohort of AFLP pregnancies.

## Sources of Funding

R.I. Neuman and A.H. van den Meiracker are supported by the Dutch Foundation Lijf en Leven.

## Disclosures

None.

## Novelty and Significance

### What Is New?

- The levels of total PlGF (placental growth factor) can be accurately predicted from free PlGF and sFlt-1 (soluble Fms-like tyrosine kinase-1) levels using the well-established mathematical formulas of drug-receptor interactions.
- Placental synthesis of PlGF is significantly increased in acute fatty liver of pregnancy (AFLP) compared with preeclampsia or hemolysis elevated liver enzymes and low platelet count syndrome.

### What Is Relevant?

- Total PlGF can now be easily calculated from already available free PlGF and sFlt-1 levels, allowing the evaluation of other groups in whom PlGF might be up- or downregulated.

### Summary

Total PlGF levels were determined in women with AFLP compared with women without preeclampsia, preeclampsia, and hemolysis elevated liver enzymes and low platelet count syndrome. Calculation of total PlGF using mathematical formulas of drug-receptor interactions was shown to be accurate and yielded similar levels to the recently described method of thermal dissociation. Although total PlGF in preeclampsia and hemolysis elevated liver enzymes and low platelet count syndrome were similar to that in uneventful pregnancies, maternal total PlGF levels in AFLP were greatly elevated compared with all groups. In AFLP, like sFlt-1, total PlGF declined massively after delivery, suggesting that in AFLP, the increased levels of total PlGF are likely placenta-derived.
